# Identifying Optimal Testing Modalities to Increase COVID-19 Testing Access in Baltimore, Maryland: Protocol for a Household Randomized Controlled Trial

**DOI:** 10.2196/68600

**Published:** 2025-10-02

**Authors:** Jessica Duchen, Alexandra K Mueller, Saifuddin Ahmed, Jamie Perin, Courtney Borsuk, Joshua Trowell, Kelly Lowensen, Steven Huettner, Andy Peytchev, Jason E Farley, Shruti H Mehta, Jacky M Jennings

**Affiliations:** 1 Center for Child and Community Research Department of Pediatrics Johns Hopkins School of Medicine Baltimore, MD United States; 2 Department of Population, Family and Reproductive Health Johns Hopkins Bloomberg School of Public Health Baltimore, MD United States; 3 Department of International Health Johns Hopkins Bloomberg School of Public Health Baltimore, MD United States; 4 Department of Epidemiology Johns Hopkins Bloomberg School of Public Health Baltimore, MD United States; 5 Center for Infectious Disease and Nursing Innovation Johns Hopkins School of Nursing Baltimore, MD United States; 6 Center for Survey Methodology Survey Research Division Research Triangle Institute International Research Triangle Park, NC United States

**Keywords:** infectious disease, SARS-CoV-2, mobile, disease control, health equity, urban health, health disparities, healthcare access

## Abstract

**Background:**

The COVID-19 pandemic disproportionately affected low-income and racial and ethnic minority populations. Testing plays a critical role in disrupting disease transmission, but complex barriers prevent optimal testing access, particularly for Black and Latinx communities. There is limited evidence regarding the optimal testing modalities to increase testing access for these populations.

**Objective:**

This study aimed to define the optimal COVID-19 testing modalities for maximizing testing acceptance, uptake, and timeliness of receipt of results.

**Methods:**

The Community Collaboration to Combat COVID-19 (C-FORWARD) trial was a household randomized comparative effectiveness trial conducted in a representative sample of an urban population. Households across 653 census block groups were sampled using a probability proportional to size approach. The primary outcome was the completion of SARS-CoV-2 or COVID-19 testing within 30 days of randomization.

**Results:**

Between February 2021 and December 2022, a total of 1083 individuals were enrolled, including 881 (81.35%) index participants and 202 (18.65%) household members. The mean age of participants was 51 (SD 18) years.Of the total sample, 43% (n=460) of participants identified as Black or African American, 48.6% (n=526) as White, and 9% (n=91) as other, including Asian, American Indian, Native Hawaiian or Pacific Islander, and multiple races; 4.8% (n=48) of participants identified as Hispanic or Latino. At the time of enrollment, 51.1% (n=553) were currently working either full time or part time, and 32.9% (n=342) of participants had an advanced degree. In total, 80% (n=809) of participants had been tested for COVID-19 previously, with 22.3% (n=179) reporting a prior positive test for COVID-19, and 86.8% (n=890) reporting receiving at least one COVID-19 vaccination before enrollment.

**Conclusions:**

Data from the C-FORWARD trial will be used to address important questions regarding COVID-19 testing acceptance and uptake in an urban population.

**International Registered Report Identifier (IRRID):**

RR1-10.2196/68600

## Introduction

### Background

The novel SARS-CoV-2 or COVID-19 emerged as a new viral infection in December 2019. Now a global pandemic, as of December 2023, COVID-19 has infected more than 100 million people in the United States and more than 1.1 million have died [1]. COVID-19–related morbidity and mortality have disproportionately affected low-income and racial and ethnic minority populations [2]. While highly effective SARS-CoV-2 vaccines are widely available, multiple challenges have prevented optimal vaccine uptake, including vaccine hesitancy and misinformation, breakthrough infections, and the emergence of new variants potentially impacting the effectiveness of the vaccine. Therefore, nonpharmaceutical interventions, such as masking, testing, and social distancing, remain critical to controlling the COVID-19 pandemic [3].

Testing plays a crucial role in preventing transmission. Early access to testing breaks infectious disease transmission chains and reduces contact between infected and susceptible persons, particularly given the high prevalence of asymptomatic and mild disease. Early testing can also reduce COVID-19 severity, as early case identification decreases the time to treatment for infected individuals [4]. Currently, there are a variety of tests available for COVID-19 diagnosis, including polymerase chain reaction (PCR), antigen tests, and serum-based antibody tests [5]. Access to testing may be limited by geographic areas with low testing access, that is, *testing deserts*. Geographic areas with low rates of access to and availability of SARS-CoV-2 testing often result from an overwhelmed supply chain and a disjointed public health system. Jurisdictions have identified testing deserts in lower-income, highly segregated areas [6]. In 2021, rapid “at-home” antigen tests became available for purchase by the public. In theory, this should make testing more accessible at the population level; however, during infection surges, tests, including “at-home” antigen tests, were often unavailable. In addition, access to at-home tests is dependent on the ability to access stores, pharmacies, or other distributors of the at-home tests, which may be difficult in areas with limited access.

**Figure 1:**
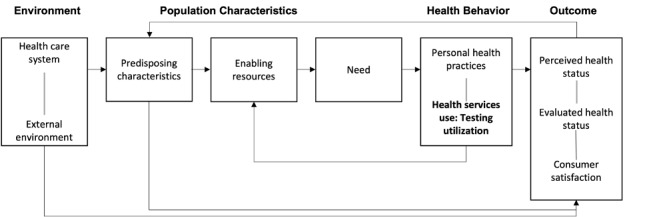
The behavioral model for vulnerable populations by Anderson: the conceptual framework of SARS-CoV-2 testing use based on the behavioral model of access to care.

There are complex multilevel barriers to optimal testing (Figure 1), and the pandemic has put into stark relief the social and structural determinants of health, leading to health inequities in testing. Evidence suggests that impoverished and minority populations experience increased barriers to testing. Latinx and Black communities are nearly 3 and 2 times more likely to be uninsured compared with non-Latinx White individuals, respectively [7]. Black individuals of all ages are also more likely to report not being able to see a doctor in the past year because of cost, which has direct implications for access to testing [8]. In addition, longstanding issues of institutional (ie, medical, research, and public health) racism resulting in mistrust and distrust, language barriers, and the cost associated with missing work all decrease the likelihood of testing among these subgroups. Exacerbating the disparity in testing access, Black individuals also experience a higher burden of disease for many chronic conditions associated with COVID-19 (eg, diabetes, hypertension, and chronic obstructive pulmonary disease), placing them at increased risk of severe COVID-19 illness and mortality [2].

### Objectives

The overall goal of the Community Collaboration to Combat COVID-19 (C-FORWARD) trial was to develop evidence related to COVID-19 testing for translation to public health control strategies. To define optimal COVID-19 testing modalities for maximizing testing acceptance, uptake, and timeliness of results, we implemented a household randomized comparative effectiveness trial in a representative sample of an urban population. This study is among the first to directly compare testing modalities in a real-world, community-based randomized trial setting, providing novel insights into how testing strategies can be optimized for public health impact. We hypothesize that, compared with the standard-of-care (SOC) arm, those randomized to the community-based, mobile van testing arm (arm 2) and the self-collection, home-based testing arm (arm 3) will be more likely to receive a COVID-19 PCR test than those assigned to the SOC arm (arm 1)*.* The secondary objectives were to determine multilevel (eg, socioeconomic and behavioral) barriers and facilitators to SARS-CoV-2 testing and to evaluate the impact of testing modality and receipt of positive results on subsequent testing behavior. The goal of this paper is to share the protocol for the C-FORWARD trial. We also present initial enrollment information.

## Methods

### Overview

**Figure 2:**
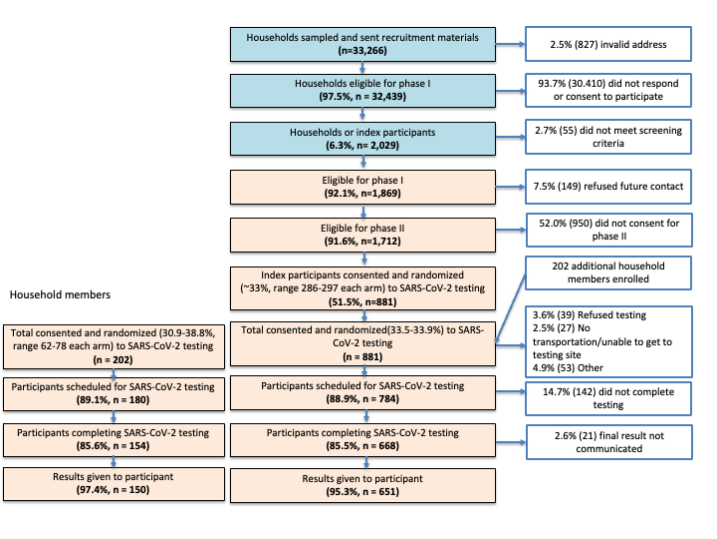
The Community Collaboration to Combat COVID-19 (C-FORWARD) project study schema.

C-FORWARD was a household randomized comparative effectiveness trial that evaluated 3 COVID-19 testing modalities to identify optimal testing modalities among a representative sample of households in Baltimore City, Maryland. The trial was conducted from February 2021 to June 2023. Figure 2 presents the C-FORWARD project study schema, illustrating the flow of enrollment from the initial survey through testing to the final trial visit.

The trial was registered at ClinicalTrials.gov (NCT04673292), and the protocol followed recommendations for reporting from the SPIRIT (Standard Protocol Items: Recommendations for Interventional Trials) 2013 statement [9]. Results will be reported in accordance with standards described in the CONSORT (Consolidated Standard of Reporting Trials) statement and CONSORT extensions for nonpharmacologic trials, cluster trials, and the draft extension for randomized controlled trials (RCTs) conducted using cohorts and routinely collected health data, which is forthcoming.

### Setting

Baltimore City is a hypersegregated city with White, Black, and Latinx residents, as well as high- and low-poverty populations, living in physically clustered, geographically isolated communities [8]. In Baltimore, Black residents have a median household income that is 54% of that of White residents. Black residents make up more than 60% of the population, yet more than twice as many Black families as White families live in liquid asset poverty, meaning they do not have sufficient savings to subsist at the poverty level for 3 months in the absence of income [9].

### Ethical Considerations

This study was approved by the Johns Hopkins University Institutional Review Board under IRB00250298. Trial staff obtained informed consent and conducted surveys for each participant in their preferred language (English or Spanish), with assent obtained from capable children as necessary and adhered to local, national, regional, and international laws and regulations regarding protection of personal information, privacy, and human rights. Participants were compensated $50 for participation in the trial.

### Study Population and Sampling Strategy

The target population included English- and Spanish-speaking households residing in 653 census block groups (CBGs), which we refer to as neighborhoods. We estimated that the target population comprised approximately 593,490 persons and 238,436 households living in the 653 CBGs in 2020.

**Figure 3:**
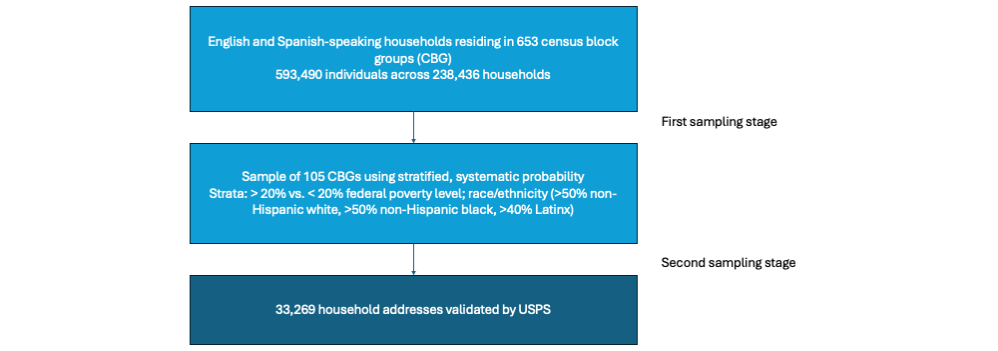
Sampling strategy for the Community Collaboration to Combat COVID-19 (C-FORWARD) trial. CBG: census block group; USPS: US Postal Service.

**Figure 4:**
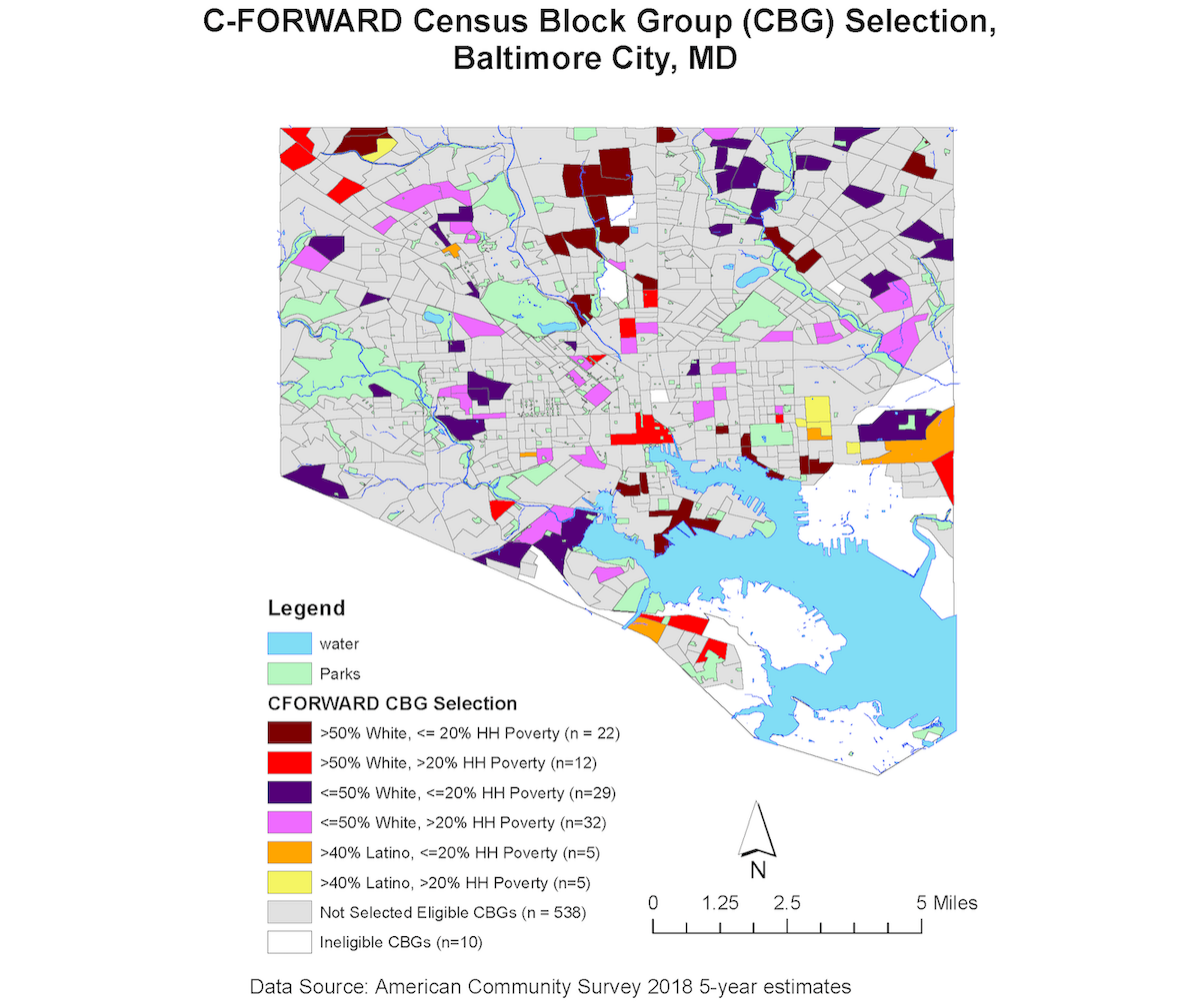
Map of selected census block groups in Baltimore City. C-FORWARD: Community Collaboration to Combat COVID-19; CBG: census block group; HH: household.

The sampling selection for the study was conducted in 2 stages (Figure 3). In the first stage, the 653 CBGs in Baltimore City were placed into 3 primary strata by race and ethnicity (>50% non-Hispanic White, >50% non-Hispanic Black, and >40% Latinx) and then additionally stratified by the percentage of households below the US federal poverty line (>20% and £20%) to create 6 strata. The strata were created to optimize the selection of each population subgroup. A sample of 105 CBGs was then selected using a stratified, systematic probability proportional to size sampling strategy, where size was defined by the estimated number of eligible households (Figure 4). Strata and household estimates for CBGs were generated using the 2018 American Community Survey.

In the second sampling stage, a total of 33,269 household addresses were provided by a vendor who receives updated sampling annually with US Postal Service data on valid household addresses. Household addresses were validated using US Postal Service data. Of the 33,269 households in the second-stage sampling frame, 7.5% (2495) were fielded, and of these, 96.6% (2411) of the households were successfully screened. During the screening, 93% (2243) had at least 1 English- or Spanish-speaking person aged between 18 and 99 years.

### Inclusion and Exclusion Criteria

Textbox 1 presents the inclusion and exclusion criteria for the C-FORWARD participants. Households had to be among the selected addresses in Baltimore City and have at least 1 household member aged ≥18 years who spoke English or Spanish and was psychologically fit to complete the survey (ie, the household index). The first household member aged ≥18 years to consent and enroll in the study was defined as the household index. After enrollment and completion of the baseline survey, the index was invited to enroll in the RCT, along with any other eligible household members.

**Recruitment.**

**Inclusion criteria for households**

1. Selected address within Baltimore City
2. At least 1 member of the household aged >18 years who speaks English or Spanish
3. At least 1 member of the household provides informed consent
4. At least 1 member of the household who is psychologically fit to complete the survey

**Exclusion criteria for households**

1. An adult member of the household is under the influence of illicit substances, in the opinion of the phone interviewer
2. Residence in a nursing home, halfway house, or shelter
3. Psychologically unfit to complete the survey
4. Not a selected household address

**Inclusion criteria for individuals**

1. Reports primary residence within the sampled household
2. Provides informed consent
3. For children aged ≥12 years, provides assent
4. Aged >5 years
5. Speaks English or Spanish

**Exclusion criteria for individuals**

1. The person providing informed consent is under the influence of illicit substances.

Additional individual household members who enrolled in the RCT were aged ≥5 years, reported residence within the sampled household, spoke English or Spanish, and resided within the household of an index participant. Individual household members were excluded if they were residents of a nursing home, halfway house, shelter, or if they did not reside at the selected household address. Individuals who were psychologically unfit to complete surveys, under the influence of an illicit substance, or aged <5 years were also excluded.

Recruitment for households involved a door hanger placed at the household, as well as a postcard and 3 separate letters sent by mail with study information and a link to complete an online screening. Participants were able to complete the screening and survey in multiple ways, including online, over the phone, or through the mail. Household members who completed the online screening and provided a phone number were contacted by trial staff via phone. They then had the option to provide informed consent over the phone. After consent, the baseline survey was completed by a trial staff member over the phone. A copy of the informed consent and baseline survey was also included in each letter mailed to households, along with a stamped and preaddressed envelope to return the completed consent and baseline survey.

All recruitment materials (ie, website, postcards, letters, and door hangers), trial documents (ie, informed consent forms and surveys), and patient education materials were offered in English and Spanish. For Spanish-speaking participants, all trial visits and communications (eg, visit scheduling, appointment reminders, and results review) were offered in Spanish.

### Informed Consent and Enrollment

One individual from each household (the index) consented and was enrolled. For Spanish-speaking individuals, all consent and assent documents were translated and certified by certified translators, and communication was conducted in Spanish by certified translators. At the time of consent, the household was randomized to 1 of 3 testing arms, and the testing visit was scheduled.

All index household participants were eligible to enroll in the RCT. Research staff also asked for permission to contact other household members (identified in the household inventory) to ascertain their interest and willingness to participate in the trial. For willing households, research staff collected contact information for all household members and documented the permission to contact. Unwilling households were informed that other household members could be referred to the trial at any time.

RCT enrollment for the household index either continued immediately after completion of the survey (if the participant was willing) or at a later mutually agreed upon day and time. The informed consent form for the RCT was provided to the household index participant via a website address or link (sent online via text or email). If the index participant was interested in enrolling in the RCT but did not have access to the internet, a hard copy of the consent was mailed, and enrollment took place at a later date. After receipt of the consent form, the trial staff member reviewed the consent form via the phone to ensure the participant provided informed consent. The trial staff member electronically signed the consent form to document oral consent for (1) participation in the RCT; (2) storage of biospecimens; (3) additional genetic studies; (4) future contact; (5) medical record review through the Chesapeake Regional Information System for Patients; and (6) sharing zip code with the Duke Clinical Research Institute.

The same consent form shared with the household index was also provided to all other household members aged ≥18 years. The same procedures followed to obtain informed consent during the survey were followed. RCT enrollment for other individual household participants included completion of a survey, while RCT participation for all household participants included testing according to the household’s randomization assignment (see Study Arms), collection of host genetic material, and storage of biospecimens. In addition, each participant was provided with the phone numbers of the principal investigator and the Institutional Review Board office to contact if they had additional questions or concerns.

### Questionnaires

Participants were asked about sociodemographic and health-related information through self-administered online or phone-administered surveys, depending on participant preference. Sociodemographic characteristics included age, race, and ethnicity, sexual orientation, education, employment status, and relationship status. Health-related information included the presence of chronic health conditions and health care–seeking behaviors. Questions were also included on social distancing and COVID-19 prevention behaviors, as well as previous COVID-19 symptoms, testing and results, and vaccination.

### Randomization

Randomization occurred at the household level. Once any adult household member was enrolled in the RCT, randomization to 1 of the 3 testing modalities was performed using an individual-level stratified blocked randomization approach. First, the sampled CBGs (n=105) were placed into 12 geographic groups based on geographic location. Within each of these 12 geographic groups, stratum-specific allocation sequences were generated based on race and ethnicity (non-Hispanic White, including non-Hispanic Other, non-Hispanic Black, and Hispanic/Latinx) and the percentage of households below the US federal poverty line (>20% vs £20%), resulting in a total of 96 strata. The sequences were integrated into REDCap (Research Electronic Data Capture; Vanderbilt University) using varying block sizes of 3 and 6, and households were allocated at a 1:1:1 ratio to each testing modality. Varying block sizes were used to minimize the risk of selection bias. Household-level randomization applied to all members of the household who agreed to participate in the RCT. Randomization assignment was made by REDCap, with study team members unaware of the previous assignments, ensuring allocation concealment.

### Study Arms

**Figure 5:**
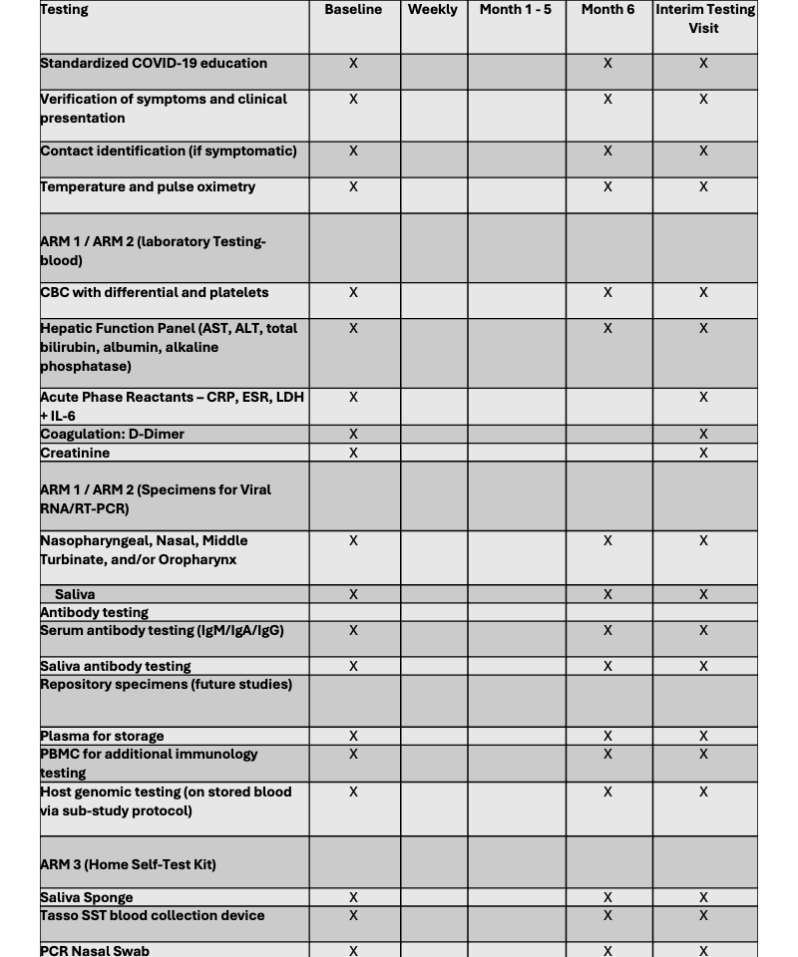
Biological samples and laboratory testing by the trial arm.

The enrollment target was 1386 households, randomized 1:1:1 to 1 of 3 SARS-CoV-2 PCR and antibody testing modalities (Table 1): arm 1, fixed ambulatory outdoor testing site (SOC testing); arm 2, community-based mobile van testing; or arm 3, self-collected, home-based testing. A full description of the tests is listed in Figure 5.

**Table 1:** Summary of testing modalities.

Testing modality	Location	Modality scheduling differences	Modality logistical differences	Laboratory sample type
Arm 1: fixed site, standard-of-care	Johns Hopkins Health System testing sites	Participants selected 1 of 3 sites and followed the appointment schedule.	Set time and fixed site, but the location and schedule may be inconvenient	Nasal swab, NP^a^ swab, saliva, and venipuncture
Arm 2: mobile van	Central location within CBG^b^	Participants arranged testing at a single testing van site in their CBG, at any time of the day (no fixed time).	Crowding and long waits if many individuals arrive at the same time; highly convenient	Nasal swab, NP swab, saliva, and venipuncture
Arm 3: home-based self-collection	Participants’ home	Nasal swab and saliva were delivered to participants via courier service; participants completed 1 shipment for the 2 packages that would be sent back.	Most convenient, but self-sampling may result in specimen errors; delay or failure to return the sample by mail is costly and leads to no results	Nasal swab and saliva; for participants enrolled before December 10, 2021: blood self-collection device

^a^NP: nasopharyngeal.

^b^CBG: census block group.

#### Fixed Site (Arm 1)

This arm was designed to replicate SOC and included all 3 Johns Hopkins Medical Institution ambulatory outdoor testing sites across Baltimore City. Each testing site used a traditional appointment-based scheduling system. Participants were given a choice of 1 of 3 outdoor testing locations based on their preferences. Trial staff scheduled appointments for testing based on testing availability and participant schedule.

#### Community-Based Mobile Van Testing (Arm 2)

This arm offered the convenience of highly accessible testing, close geographic proximity, and the flexibility of no fixed appointment time. Each of the 12 geographic strata (see the Randomization section) had a single, centrally located testing site within the area, providing similar geographic access across households.

#### Home-Based Testing (Arm 3)

The individuals in this arm received a home-based testing kit delivered to the household by a courier service. The testing kit included a Becton Dickinson swab for SARS-CoV-2 PCR testing, a Tasso device for the collection of 5 mL of blood, and an Oracol swab for saliva collection. Easy-to-use printed instructions were included in English and Spanish. In addition, the participants randomized to home-based collection had the option to view prerecorded videos demonstrating correct collection techniques or to schedule virtual “on-demand” coaching sessions with members of the study team. If a sample was insufficient or collected incorrectly, an additional testing kit was provided, along with a phone call from a study team member detailing the correct collection method. After completing specimen collection, participants called a study team member to arrange for specimen pick-up within 24 hours of collection.

### Study Changes

Because of the unprecedented and ever-evolving nature of the COVID-19 pandemic, several changes to study design were implemented over the course of the study. These changes were necessary to ensure continuation of the trial, and we do not believe they impacted the validity of the trial. The changes are summarized in Table 2.

**Table 2:** Study changes implemented during the Community Collaboration to Combat COVID-19 (C-FORWARD) trial.

Change	Date implemented	Affected testing modalities	Description
Change from 3 to 2 fixed testing sites	June 2021	Arm 1: fixed site, standard-of-care	One Johns Hopkins Health System testing site closed in June 2021, resulting in 2 fixed testing sites instead of 3
Removal of the blood self-collection device for home-based collection	December 2021	Arm 3: home-based self-collection	The Tasso blood self-collection device was removed from the home-based testing, eliminating serum-based antibody testing from the participants in this arm.
Change in length of follow-up from 12 to 6 months	January 2022	All study arms	The length of follow-up for the study was changed from 12 to 6 months, reducing the total number of possible monthly visits from 12 to 6.
Change from self-administered swab packaged by and returned to Johns Hopkins laboratory to Everlywell home testing kit	January 2022	Arm 3 home-based self-collection	The arm 3 home testing kit was changed from a self-administered swab returned to the John Hopkins laboratory to a PCR^a^ kit with provisions for shipping samples directly to a company called Everlywell, via a UPS^b^ dropbox or fixed UPS location.
Change in household member eligibility from age 5 to 16 years	January 2022	All study arms	Because of the switch to the Everlywell testing kit, participants aged <16 years could not be tested using the home-based collection. Because of this, eligibility criteria across all study arms were updated to 16 years.

^a^PCR: polymerase chain reaction.

^b^UPS: United Parcel Service.

### Study Outcomes

The primary outcome was completion of SARS-CoV-2 testing, defined as the proportion of RCT-consented participants who completed SARS-CoV-2 testing within 30 days of consent or randomization (Table 3). Completion of SARS-CoV-2 testing was defined as completion of SARS-CoV-2 PCR testing at the testing site (for the fixed-site arm and mobile van arm) or return of the PCR swab by the participant to the laboratory via courier service. Completion of testing was verified and documented by laboratory staff. After consent, participants were scheduled for a testing visit at their assigned testing modality. Trial staff made up to 10 attempts to schedule each participant for testing. The participants were allowed to reschedule their testing visit up to 3 times.

Secondary outcomes (Table 3) included time (in days) to completion of SARS-CoV-2 PCR testing, receipt of SARS-CoV-2 PCR test results within 30 days of consent, and time from SARS-CoV-2 PCR test to receipt of SARS-CoV-2 PCR test results among those who received test results. SARS-CoV-2 results were communicated to participants by trial staff or through EMR messaging (Epic MyChart). Time to completion of SARS-CoV-2 PCR testing was defined as the number of days from the date of consent or randomization to the date of SARS-CoV-2 PCR testing at the testing site (for the fixed-site arm and mobile van arm) or the return of the PCR swab to the laboratory. Time to receipt of results was measured as the number of days from the date of consent or randomization to the date of SARS-CoV-2 PCR test results were communicated to the participant and documented by trial staff or through EMR messaging (Epic MyChart). Knowing one’s COVID-19 status—whether positive or negative—is essential for determining the need to isolate or notify close contacts of potential exposure, thereby helping to interrupt the chain of transmission.

**Table 3:** Primary and secondary outcomes for the Community Collaboration to Combat COVID-19 (C-FORWARD) trial.

Outcome	Primary or secondary	Definition
Completion of SARS-CoV-2 testing	Primary	The proportion of RCT^a^-consented participants who complete SARS-CoV-2 testing within 30 days of consent or randomization. Completion of SARS-CoV-2 testing was defined as the completion of SARS-CoV-2 PCR^b^ testing at a testing site (for the fixed-site arm and mobile van arm) or the return of a PCR swab by the participant to the laboratory via courier service. Completion was verified by laboratory staff.
Time (in days) to completion of SARS-CoV-2 PCR testing	Secondary	Number of days from the date of consent or randomization to the date of SARS-CoV-2 PCR testing at the testing site (for the fixed-site arm and mobile van arm) or return of the PCR swab to the laboratory.
Time (in days) from SARS-CoV-2 PCR test to receipt of SARS-CoV-2 PCR test results among those who received test results	Secondary	Number of days from the date of consent or randomization to the date SARS-CoV-2 PCR test results were communicated to the participant and documented by trial staff or through EMR^c^ messaging (Epic MyChart).

^a^RCT: randomized controlled trial.

^b^PCR: polymerase chain reaction.

^c^EMR: electronic medical record.

### Sample Size and Power Considerations

We calculated power for comparing the primary outcome related to randomization (the proportion of participants who completed SARS-CoV-2 testing) across arms (arm 3 vs arm 1 and arm 2 vs arm 1). Given that we enrolled 1386 households across the 3 arms, and that randomization occurred at the household level, we were powered to detect a difference in the level of testing in arms 2 or 3 and the control arm (arm 1) of approximately 11%, (from 50% in the control arm to 61% in the comparison arm) with at least 80% power. We assumed an overall type 1 error of 0.05 and controlled for multiple comparisons using a Bonferroni correction. Because of the possibility of intrahousehold correlation—meaning testing among individuals in the same household may be highly correlated—we conservatively assumed that each household enrolled only 1 individual. This detectable difference of 11% corresponds to a prevalence ratio of 1.2 when testing prevalence in the control arm is 50%, and as low as 1.17 if the testing prevalence in the control arm is 70%. We assumed a baseline testing prevalence of 50% in the control arm in the absence of pilot data, based on random chance.

### Planned Analyses

We hypothesize that, compared with the SOC arm, participants randomized to the community-based mobile van testing arm (arm 2) and the self-collection home-based testing arm (arm 3) will be more likely to receive a COVID-19 PCR test than those assigned to the SOC arm (arm 1)*.*

#### Primary Analyses

The primary analysis will use an intention-to-treat approach, limited to index participants. To mitigate the effects of potential intrahousehold correlation, the primary analysis will include only the index household member. Poisson regression will be used to calculate prevalence ratios comparing the effect of each of the 2 testing modalities (mobile van testing and home-based testing) with the SOC arm (fixed-site testing) on the primary outcome of interest—the proportion of participants who received a test within 30 days. Because of randomization, we assume that factors salient to testing access will be balanced across arms and proportions will be directly comparable without adjustment. However, we will assess whether confounding by key baseline factors is present (eg, age, sex, and race and ethnicity) and will complete adjusted analyses accordingly.

#### Secondary Analyses

In addition to comparing the proportion of index participants who received a SARS-CoV-2 PCR test within 30 days, we will examine the time to first SARS-CoV-2 PCR test using time-to-event analysis among index participants (1 per household). We will measure the time from enrollment to the first testing event for each participant.

Each participant’s time until testing will be censored when the participant exits the study. We will use Cox proportional hazards regression to compare the rate of testing between arms and to assess whether participants in the mobile testing arm (arm 2) or the home-based testing arm (arm 3) tend to be tested earlier or later than those in the SOC arm. As in the primary analysis, we expect that randomization will result in comparable characteristics across arms; however, we will consider adjusting for demographic factors or characteristics that are not balanced across arms.

We will also examine the secondary outcome of receipt of SARS-CoV-2 PCR test results within 30 days of the RCT consent, expressed as a percentage of trial participants in each arm. To compare this secondary outcome between arms, we will use Poisson regression to estimate the prevalence ratios for arms 2 and 3 relative to the SOC arm (arm 1). Consistent with our analysis of the primary outcome, we will adjust for baseline factors if they appear different across the arms at baseline.

We will examine 2 additional secondary outcomes among study participants: (1) time from SARS-CoV-2 PCR test to receipt of SARS-CoV-2 PCR test results and (2) time from consent to receipt of SARS-CoV-2 PCR test results. Both outcomes will be compared across study arms using time-to-event analysis with Cox proportional hazards regression, consistent with the analysis of time from enrollment to the first SARS-CoV-2 PCR test. Participants who do not complete a SARS-COV-2 PCR test during the study period will still contribute to the analyses of these secondary outcomes as having censored event times. Therefore, this analysis will include all participants.

We will also examine effect modification for a selection of demographic characteristics by repeating the analysis of the primary and secondary outcomes as specified earlier within specific strata, including (1) race and ethnicity (Black, Latinx, and White); (2) gender; (3) neighborhood poverty (households in CBG with >20% of households below the federal poverty line and households in CBG with <20% of households below the federal poverty line); (4) household poverty; (5) essential worker status; (6) exposure or symptoms; (7) vaccination; (8) age; (9) education (high school diploma or not); and (10) household size (using the median as the threshold).

Primary and secondary analyses will be conducted among the index participants, so that only 1 participant per household is included. We will repeat these analyses, including all participants, accounting for clustering within the household using generalized estimating equations for Poisson regression and a robust variance estimator for Cox proportional hazards regression.

Household members may differ appreciably from the index participants because they are enrolled after randomization is complete, and thus their decision to participate and motivation to undergo testing may be influenced by the randomization arm. Thus, additional secondary outcomes will examine testing uptake and completion among household members only.

#### Time-Stratified Analyses

To account for temporal changes and the evolving pandemic landscape throughout the study period, primary and secondary analyses will be stratified by time. Because of the availability of at-home rapid testing kits, the Omicron variant surge, and the changes in the home kit testing brand in the home collection arm, analyses will be stratified by enrollment before February 1, 2021, and after February 1, 2021.

## Results

C-FORWARD enrollment began on February 25, 2021, and concluded on November 23, 2022. A total of 1083 individuals were enrolled, including 881 (81.35%) index participants and 202 (18.65%) household members. Baseline characteristics are reported in Table 4. The mean age of participants was 51 (SD 18) years, with a range of 5 to 98 years. Overall, 42.5% (n=460) of the participants identified as Black or African American, 48.6% (n=526) as White, 3.4% (n=37) as multiple races, 2.7% (n=29) as Asian, and 2.9% (n=31) as another race, while 4.8% (n=48) identified as Hispanic or Latino. Most participants (n=553, 51.1%) were currently working either full time or part time at the time of enrollment. Approximately 32.9% (n=342) of participants had an advanced degree.

At the time of enrollment, over three-quarters (n=809, 79.9%) of participants had been tested for COVID-19 previously, with 22.3% (n=179) reporting a prior positive COVID-19 test. Furthermore, 86.8% (n=890) of participants reported receiving at least 1 COVID-19 vaccination before enrollment.

**Table 4:** Selected baseline characteristics of participants enrolled in Community Collaboration to Combat COVID-19 (C-FORWARD; N=1083).

Characteristics		Population (N=1083)
Households, n		881
Age (y), mean (SD)		51 (18)
Sex (male), n (%)		380 (35.1)
**Race or ethnicity, n (%)**		
	Asian	29 (2.7)
	Black or African American	460 (42.5)
	Hispanic or Latino	48 (4.8)
	Multiple races	37 (3.4)
	Other	31 (2.9)
	White	526 (48.6)
**Employment,** **n (%)**		
	Working now (either full time or part time)	553 (51.1)
	Retired, disabled, or keeping house	384 (35.5)
	Student	64 (5.9)
	Looking for work, unemployed	53 (4.9)
	Unknown	29 (2.7)
**Essential worker (n=972), n (%)**		
	Among enrolled adults with responses	241 (24.8)
**Educational attainment (n=1038), n (%)**		
	High school graduate or less	264 (25.4)
	Some college level, technical, vocational degree, or associate’s degree	223 (21.5)
	Bachelor’s degree	201 (19.4)
	Other advanced degree	342 (32.9)
**Health insurance (n=854), n (%)**		
	No health insurance	29 (3.4)
	Public (Medicare, Medicaid, and Tricare)	376 (44)
	Private (purchased directly or through employment)	420 (49.2)
Ever been tested for COVID-19? (Self-report, n=1013), n (%)		809 (79.9)
Ever tested positive for COVID-19? (Self-report, n=804)		179 (22.3)
COVID-19 vaccination (self-report, n=1025), at least 1 dose, n (%)		890 (86.8)

## Discussion

### Anticipated Findings

This trial will provide critical information on the best practices for SARS-CoV-2 testing in communities with a high prevalence of vulnerable populations. Beyond increasing our understanding of SARS-CoV-2 testing, this study will help to characterize the prevalence of COVID-19 infection. Even with expanding access to pharmaceutical interventions, COVID-19 testing remains crucial to control the spread of infection. Testing strategies must be well studied to determine the most effective and accessible strategies, inform public policy, and public health prevention and control activities. The C-FORWARD trial will provide critically needed insights into optimal testing strategies, particularly for vulnerable populations most affected by the pandemic.

### Limitations

Several implementation-related challenges were encountered during the C-FORWARD trial. Given the ever-changing COVID-19 pandemic landscape and region-specific surges in COVID-19 incidence, testing demand was often both sporadic and urgent. The emergence of new variants also resulted in the current chaotic SOC, which varied by testing location, even within the same city, making it difficult to have a true comparison arm.

Temporal trends in the delivery approach of the SOC over the duration of the trial affected our ability to draw comparisons between the fixed site and other testing modalities. However, our ongoing ethnographic work and community engagement will capture these changes so that we can address them in the analysis as appropriate (eg, stratifying by time). It was also possible that randomized households or individuals sought testing outside of the study.

Furthermore, responses to surveys are subject to social desirability and recall bias; moreover, recall may be influenced by prior COVID-19 infection status, which is one of the key exposures of interest. We will use 2-week time frames for most behaviors to minimize this effect. Loss to follow-up is another concern, particularly as attitudes toward COVID-19 change and fatigue with study participation sets in. Individuals across all testing modalities may have sought testing outside of the study, introducing the risk of contamination across study arms. To mitigate this risk, the enrolled individuals were scheduled for testing as soon as possible after enrollment and randomization, and testing outside the study was assessed through surveys at baseline and monthly follow-up visits.

While our findings will provide valuable insights, the generalizability of the results may be limited to hypersegregated urban areas similar to Baltimore City. However, identifying optimal testing strategies that increase access to testing in a pandemic setting is an important consideration for all public health officials, particularly for communities with limited access to traditional health care settings. Individuals in rural areas may be at heightened risk for chronic health conditions that worsen COVID-19–associated morbidity and mortality, along with having farther distance to travel and reduced access to health care settings [10,11]. For example, rural areas could benefit from mobile testing vans or increased availability of tests that can be completed and returned via mail.

### Conclusions

As early and accessible testing can prevent forward transmission and improve individual-level outcomes, this trial aims to identify the most effective approach to providing access to COVID-19 testing. In addition to improving our understanding of how testing modality affects access and the effectiveness of testing initiatives, this work focuses on access within populations with the highest burden of COVID-19 disease, and thus those who will benefit most from these findings. The framework established through this trial can be adapted to other infectious diseases—such as influenza, respiratory syncytial virus, or emerging respiratory viruses—by evaluating testing access, uptake, and outcomes in similarly vulnerable populations. Leveraging these insights across disease areas can support timely public health responses and equitable diagnostic strategies.

## Abbreviations

C-FORWARD: Community Collaboration to Combat COVID-19
CBG: census block group
CONSORT: Consolidated Standards of Reporting Trials
PCR: polymerase chain reaction
RCT: randomized controlled trial
REDCap: Research Electronic Data Capture
SOC: standard-of-care
SPIRIT: Standard Protocol Items: Recommendations for Interventional Trials

## Data Availability

The data that support the findings of this study are available from the corresponding author upon reasonable request.
